# A decade-long study demonstrates that a population of invasive sea lamprey (*Petromyzon marinus*) can be controlled by introducing sterilized males

**DOI:** 10.1038/s41598-024-61460-1

**Published:** 2024-06-03

**Authors:** Nicholas S. Johnson, Sean A. Lewandoski, Aaron K. Jubar, Matthew J. Symbal, Benson M. Solomon, Gale A. Bravener, Jessica M. Barber, Michael J. Siefkes

**Affiliations:** 1 U.S. Geological Survey, Great Lakes Science Center, Hammond Bay Biological Station, 11188 Ray Road, Millersburg, MI 49759 USA; 2 U.S. Fish Wildlife Service, Marquette Biological Station, 1095 Cornerstone Drive, Marquette, MI 49855 USA; 3 U.S. Fish and Wildlife, Ludington Biological Station, 5050 Commerce Drive, Ludington, MI 49660 USA; 4 Fisheries and Oceans Canada, Sea Lamprey Control Centre, 1219 Queen Street East, Sault Ste., Marie, ON P6A 2E5 Canada; 5 Great Lakes Fishery Commission, 2200 Commonwealth Blvd., Suite 100, Ann Arbor, MI 48105 USA

**Keywords:** Ecology, Conservation biology, Invasive species, Environmental sciences

## Abstract

The release of sterilized insects to control pest populations has been used successfully during the past 6 decades, but application of the method in vertebrates has largely been overlooked or met with failure. Here, we demonstrate for the first time in fish, that a small population of sea lamprey (*Petromyzon marinus*; Class Agnatha), arguably one of the most impactful invasive fish in the world, can be controlled by the release of sterilized males. Specifically, the release of high numbers of sterile males (~ 1000's) into a geographically isolated population of adult sea lamprey resulted in the first multiyear delay in pesticide treatment since treatments began during 1966. Estimates of percent reduction in recruitment of age-1 sea lamprey due to sterile male release ranged from 7 to 99.9% with the precision of the estimate being low because of substantial year-to-year variability in larval density and distribution. Additional monitoring that accounts for recruitment variability in time and space would reduce uncertainty in the degree to which sterile male release reduces recruitment rates. The results are relevant to vertebrate pest control programs worldwide, especially as technical opportunities to sterilize vertebrates and manipulate sex ratios expand.

## Introduction

Controlling or eradicating insect pests through the release of sterilized conspecifics was independently conceived by three researchers in disparate parts of the world during the 1940s^1^. Success of the technique relies on at least four conditions: (1) sterilized animals are equally competitive in mating as wild individuals, (2) sterile individuals are released during the time and at the location wild individuals are mating, (3) production of offspring decreases in a predictable way when the effective number of reproducing individuals decrease (population growth is density dependent), and (4) sterilized individuals are released at great enough numbers to overwhelm mating among wild individuals (overflooding ratio)^2^. These conditions have been met for many pest insects like screw worm (*Cochliomyia hominivorax*), the Mediterranean fruit fly (*Ceratitis capitata*), and pink bollworm (*Pectinophora gossypiella*; (see^3^ for a full review)) and accordingly, numerous populations have been suppressed, eradicated, contained, or establishment prevented through coordinated production and release of sterile individuals^4^.

Sterile male release may be relevant for the control of 1000's of other species of pest animals^5,6^, but the approach is infrequently considered for vertebrates given challenges related to sterilization. Efforts to develop sterilization methods that do not reduce the reproductive vigor of an individual have been investigated in amphibians^7^, crustaceans^8^, and mammals^9,10^, but, to date, have not been applied at management-relevant scales. To our knowledge, in fishes (vertebrates with fins that live in water), the only species where a sterilization method has been validated and sterilized individuals have been released to control an invasive population has been the sea lamprey (*Petromyzon marinus*).

Integrated control of sea lamprey in the North American Great Lakes (Great Lakes) has been desired since 1980^11^, but this vision has largely not been realized^12^. Sea lamprey are cartilaginous agnathan fish native to the American and European Coasts of the Atlantic Ocean where they provide valued ecological services in streams; sea lamprey clean substrate when constructing nests in gravel and provide nutrients when they die after spawning^13^. Sea lamprey larvae filter feed for several years prior to transforming to juveniles and migrating to the Ocean to parasitize fish^13^. Sea lamprey invaded the Great Lakes in the 1930s using canals built for commerce vessels^14^. Juvenile sea lamprey that parasitize freshwater fish are destructive because each sea lamprey consumes more than 1 kg of blood in the 12–18 month transition to adulthood and most freshwater fishes in the Great Lakes are too small to survive that magnitude of blood loss^15,16^. Therefore, an international program to control sea lamprey-induced mortality on valued fishes has been in place since the 1950s^17^ and reduced sea lamprey populations by 90% across all the Great Lakes^18^. However, a critical vulnerability of the control program is its complete dependence on two complementary tactics: (1) barriers that block adult sea lamprey migration thereby aggregating larval sea lamprey populations into stream segments where (2) a pesticide selective for lamprey (lampricide) can be applied^17^.

Male sea lamprey build nests along with females, males often mate with multiple females, and both sexes die after spawning^19^. Therefore, tactics that exploit the unique physiology and mating system^14^ of sea lamprey may be able to supplement the use of barriers and lampricide^12^. Following the conceptual model developed by Knipling^20^, a method for sterilizing adult male sea lamprey through injection of a chemosterilant in a laboratory was developed in 1970s by  Lee Hanson^21^. Subsequent laboratory and field tests in the 1980s, 1990s, and 2000s, demonstrated that sterilized males completed sexual maturation, arrived to spawning habitat at rates consistent with the number released, attracted and mated with females, and that embryo survival was reduced in places where sterile males spawned^22^. However, despite the experimental release of over 100,000 sterile males in Lake Superior tributaries from 1991 to 1996 and the release of ~ 410,000 sterile males in the St. Marys River from 1991 to 2011 (~ 20,000 per year), a direct connection between sterile male release, a reduction in larval sea lamprey recruitment to age-1, and a reduction in lampricide treatment was not made^23^. Given the equivocal outcomes and opportunities to expand lampricide control options, the sterile male release technique was discontinued during 2011^23^. Furthermore, a field test evaluating the efficacy of sterile female release also did not document a reduction in sea lamprey recruitment^24^.

Release of sterilized individuals for pest control has been most successful when population density is low, populations are isolated, and populations are overwhelmed with sterile individuals^4^. A numeric model of a closed sea lamprey population where 30% of the adult population was trapped with a subsequent release of a 10:1 ratio of sterile to wild males resulted in a nearly 90% reduction in abundance in a single generation^25^. However, these idealized modeled conditions were not met when sterile males were released in tributaries of Lake Superior and the St. Marys River; ratios of sterile to wild males ranged from 1:1 to 1:5 and populations were open to immigration or emigration from non-treated tributaries^23^.

**Figure 1:**
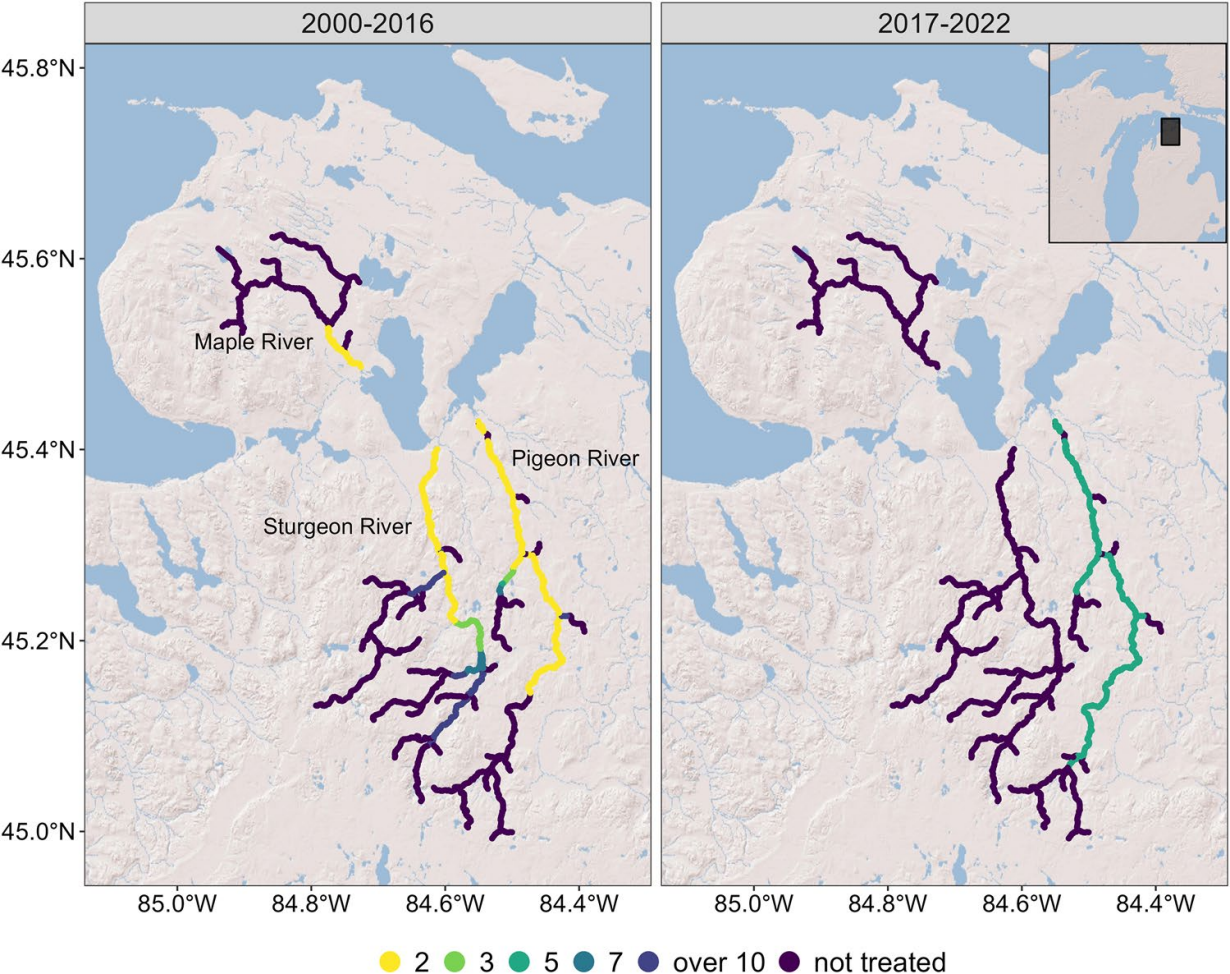
Average lampricide treatment frequency (number of years between applications) in the Pigeon, Sturgeon, and Maple Rivers during a reference period prior to sterile male release (2000–2016) and after the initiation of sterile male release (2017–2022). Sterile male release did not occur in 2020. The Sturgeon and Maple Rivers flow into Burt Lake while the Pigeon River flows into Mullett Lake. The entire watershed drains into northern Lake Huron.

In 2013, a fish biologist informed us of a population of sea lamprey that may complete its lifecycle within a tributary to Lake Huron, USA (Cheboygan River watershed). By 2016, we characterized this population that completes its lifecycle by spawning in the Pigeon, Sturgeon, and Maple Rivers and parasitizing fishes in Burt and Mullett Lakes (Michigan, U.S.A., Fig. 1;^26^). By contrast, a typical population of sea lamprey would migrate to the Great Lakes to feed on fish. The Cheboygan River population has been partially landlocked since the 1960s and has been treated with lampricide about every 4 years since 1966^27^. Although the larval population of sea lamprey can approach 1,000,000, the total adult spawning population in these three tributaries combined was estimated at less than 200 from 2013 to 2016^26^. Accordingly, the Pigeon, Sturgeon, and Maple Rivers emerged as a model system to experimentally evaluate the sterile male release technique given that the adult population was small and had low immigration rates^23^.

Here, our goal was to release sterile males in the Pigeon, Sturgeon, and Maple Rivers over multiple years at overflooding rates prescribed for the sterile insect technique (i.e. exceeding 35:1 sterile to non-sterile ratio)^28^ to determine how (1) the ratio of sterile to non-sterile males influenced the need for reoccurring pesticide treatment and (2) quantify reductions in age-1 larval sea lamprey recruitment attributed to the release of sterile males. The outcomes have direct implications for one of the largest aquatic invasive species control programs in the world and relevance for a diverse array of pest control programs worldwide.

## Results

Sterile male sea lamprey were released during 2017–2019 and 2021–2022 in the Pigeon, Sturgeon, and Maple Rivers. Ratios mostly exceeded 35 sterile males to every non-sterile male (wild male) as determined by the observed ratio of sterile to non-sterile males captured in assessment traps (Table 1; Supplemental Tables 1 and 2). However, in the Pigeon River, ratios of sterile to non-sterile males were 6:1 during 2021 and 10:1 during 2022. The lower ratios were a result of increased abundance of non-sterile males and because fewer sterile males were released (Table 1). Sterile males were not released prior to 2017 and were not released during 2020 due to the global pandemic.

**Table 1:** The number of sterile male sea lamprey released in the Pigeon, Sturgeon, and Maple Rivers, 2013–2022 (Supplemental Table 1).

River	Year	Sterile males released	Recapture number	Captured wild	Capture sterile: Normal	Abundance Est. Wild	Viable eggs before SMRT	Viable eggs after SMRT
Pigeon	2013	0	NA	2	0:1	21	535,500	535,500
Pigeon	2014	0	NA	0	0:1	NA	NA	NA
Pigeon	2015	0	NA	1	0:1	10	255,000	255,000
Pigeon	2016	0	NA	0	0:1	NA	NA	NA
Pigeon	2017	1425	413	2	413:1	8	204,000	494
Pigeon	2018	1338	428	4	224:1	12	306,000	1366
Pigeon	2019	360	72	1	144:1	5	127,500	1,118
Pigeon	2020	0	NA	0	0:1	NA	NA	NA
Pigeon	2021	275	28	9	6:1	90	2,295,000	382,500
Pigeon	2022	525	74	15	10:1	110	2,805,000	280,500
Sturgeon	2013	0	NA	1	0:1	10	255,000	255,000
Sturgeon	2014	0	NA	0	0:1	NA	NA	NA
Sturgeon	2015	0	NA	0	0:1	NA	NA	NA
Sturgeon	2016	0	NA	0	0:1	NA	NA	NA
Sturgeon	2017	1425	399	1	800:1	1	25,500	32
Sturgeon	2018	1350	257	1	514:1	5	127,500	248
Sturgeon	2019	908	163	6	54:1	33	841,500	15,583
Sturgeon	2020	0	NA	0	0:1	NA	NA	NA
Sturgeon	2021	528	23	0	> 40:1	NA	NA	NA
Sturgeon	2022	1400	70	3	35:1	60	1,530,000	43,714
Maple	2013	0	NA	1	0:1	5	127,500	127,500
Maple	2014	0	NA	2	0:1	26	663,000	663,000
Maple	2015	0	NA	2	0:1	23	586,500	586,500
Maple	2016	0	NA	0	0:1	NA	NA	NA
Maple	2017	830	498	0	> 40:1	NA	NA	NA
Maple	2018	813	203	0	> 40:1	NA	NA	NA
Maple	2019	628	44	0	> 40:1	NA	NA	NA
Maple	2020	0	NA	1	0:1	NA	NA	NA
Maple	2021	666	53	1	> 40:1	5	127,500	3188
Maple	2022	1400	70	0	> 40:1	NA	NA	NA

Sterile males and wild sea lamprey (non-sterilized and naturally in the stream) were captured in traps located in each stream (Fig. 1; Supplemental Table 2). Captured sterile males were released with a distinguishing fin-clip and captured wild sea lamprey were removed from the stream and used to estimate sex ratio and fecundity (described in^27^). The ratio of sterile males to wild males captured in traps is reported as the realized ratio of sterile to wild males. Capture rates of sterile males (Recapture Number divided by Sterile Males Released) were used to estimate wild sea lamprey abundance (Abundance Est. Wild) and given the percent female (50%) and average fecundity (51,000 eggs), an estimate of viable eggs able to be fertilized was generated. Assuming every egg in the population could be fertilized by a different male, and given the observed ratio of sterile to wild males in traps, the number of viable eggs after sterile male release (Viable Eggs Before SMRT) was estimated. SMRT, sterile male release technique.

The number of viable eggs in the study streams was estimated to decrease 83–99% during years sterile males were released. The number of viable eggs was calculated given the number of sterile males released and estimates of adult sea lamprey abundance, sex ratio, and fecundity in assessment traps. Abundance estimates of non-sterile adult female and male sea lamprey in the Pigeon, Sturgeon, and Maple Rivers were consistently less than 60 per stream each year (Table 1). The exception was for the Pigeon River where abundance estimates were about 100 during 2021 and 2022 (Table 1). As reported in a previous publication^27^, males represented 50% of the wild population and average female fecundity was 51,000 eggs (range 3100–72,000). Therefore, the estimated total number of viable eggs in each stream during years when sterile males were not released was between 127,500 and 2,800,000 (Table 1). During years when sterile males were released, the total estimated number of viable eggs after accounting for sterile males and assuming completely random mating was between 32 and 383,500 (83–99% reduction in viable eggs) .

We surveyed sea lamprey spawning locations in the Pigeon, Sturgeon, and Maple Rivers and observed sterile males and their nests throughout historically infested reaches, indicating that sterile males moved upstream and occupied spawning habitat similar to non-sterile sea lamprey. Specifically, sea lamprey spawning habitat was surveyed^22^ throughout the Pigeon, Sturgeon, and Maple Rivers during the 2017 and 2018 spawning season. Only sterile males were observed on nests in the Pigeon, Sturgeon, and Maple Rivers. No viable embryos or recently hatched larvae were collected when nests were disturbed and plankton nets were placed immediately downstream of the nest^22^. In the Pigeon River, a total of 38 sea lamprey nests were located, 12 sterile males were observed on those nests, and no sea lamprey embryos were recovered. In the Sturgeon River, a total of 10 sea lamprey nests were located, six sterile males were observed on those nests, and no sea lamprey embryos were recovered. In the Maple River, 287 sea lamprey nests were located, 172 sterile males were observed on those nests, and sea lamprey embryos were recovered from four nests, but were dead (number of embryos in each nest = 2, 3, 12, 24).

During the years when sterile males were released only the Pigeon River was selected for lampricide treatment by the Great Lakes Fishery Commission because estimates of the abundance of larval sea lamprey over 100 mm exceeded economic thresholds for treatment (Fig. 1)^29^. During the 17-year period prior to the release of sterile males (2000–2016) and using the same treatment thresholds, the Pigeon, Sturgeon, and Maple Rivers were selected for lampricide treatment 6, 5, and 5 times, respectively (described in^27^; treatments occurring roughly every 3 years).

While we found that sterile male release coincided with a decline in the abundance of larvae below an economic threshold requiring lampricide treatment, we also wanted to investigate if the abundance and distribution of age-1 sea lamprey also decreased predictably the year after sterile males were released. To do so, age-1 sea lamprey larval densities were monitored using standard electrofishing practices during years prior to and concurrent with sterilized male release in our study streams. A Bayesian statistical model was developed to investigate if changes in age-1 abundance were correlated to the number of sterile males released.

**Figure 2:**
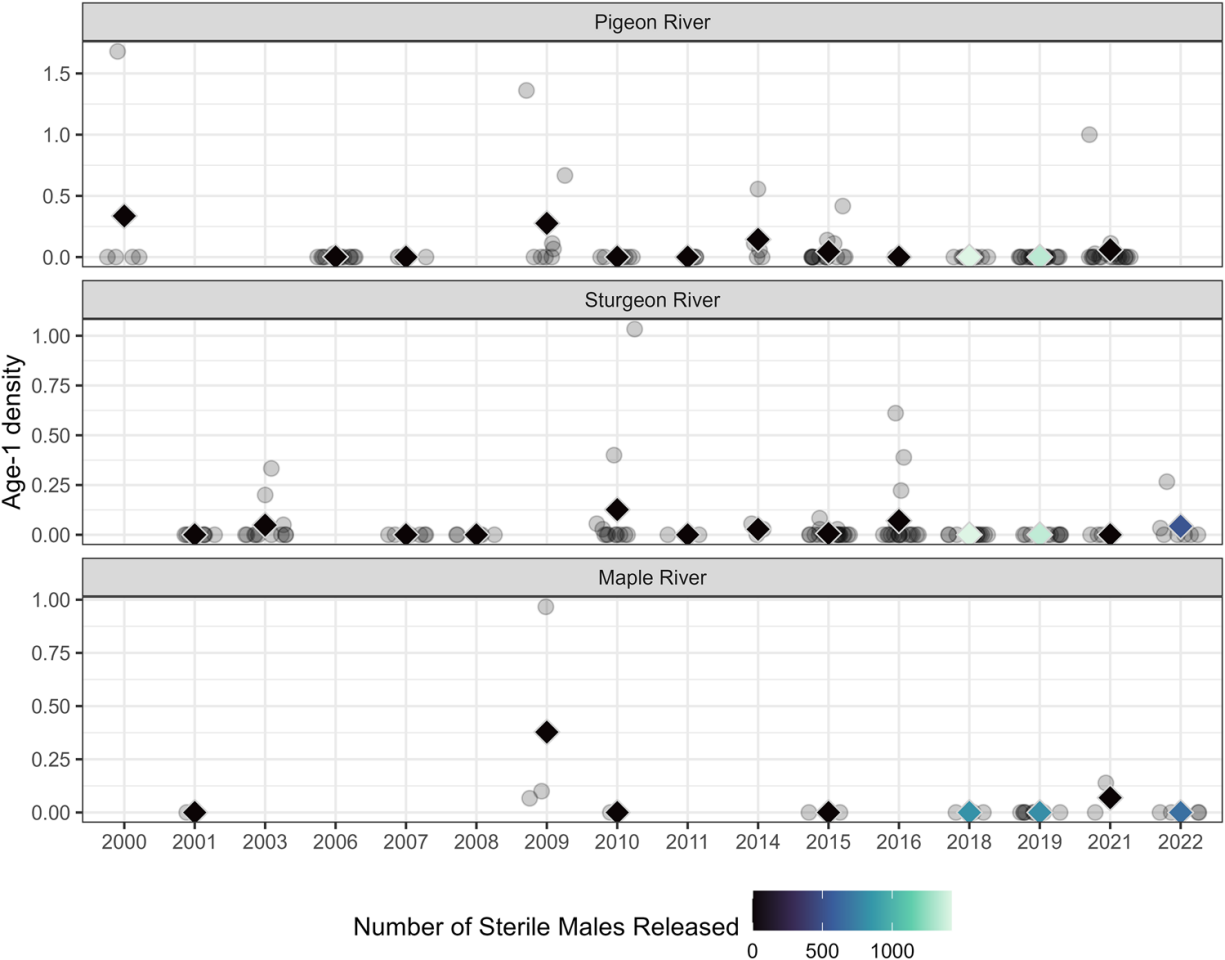
Average observed age-1 larval density (color-coded diamond symbols) and individual observations (grey circles) by year and stream. Color indicates the number of sterile male sea lamprey released during the corresponding recruitment year of the observed age-1 year class (for example—data listed under 2018 are larval that were spawned in 2017 and recruited to age-1 in 2018).

**Figure 3:**
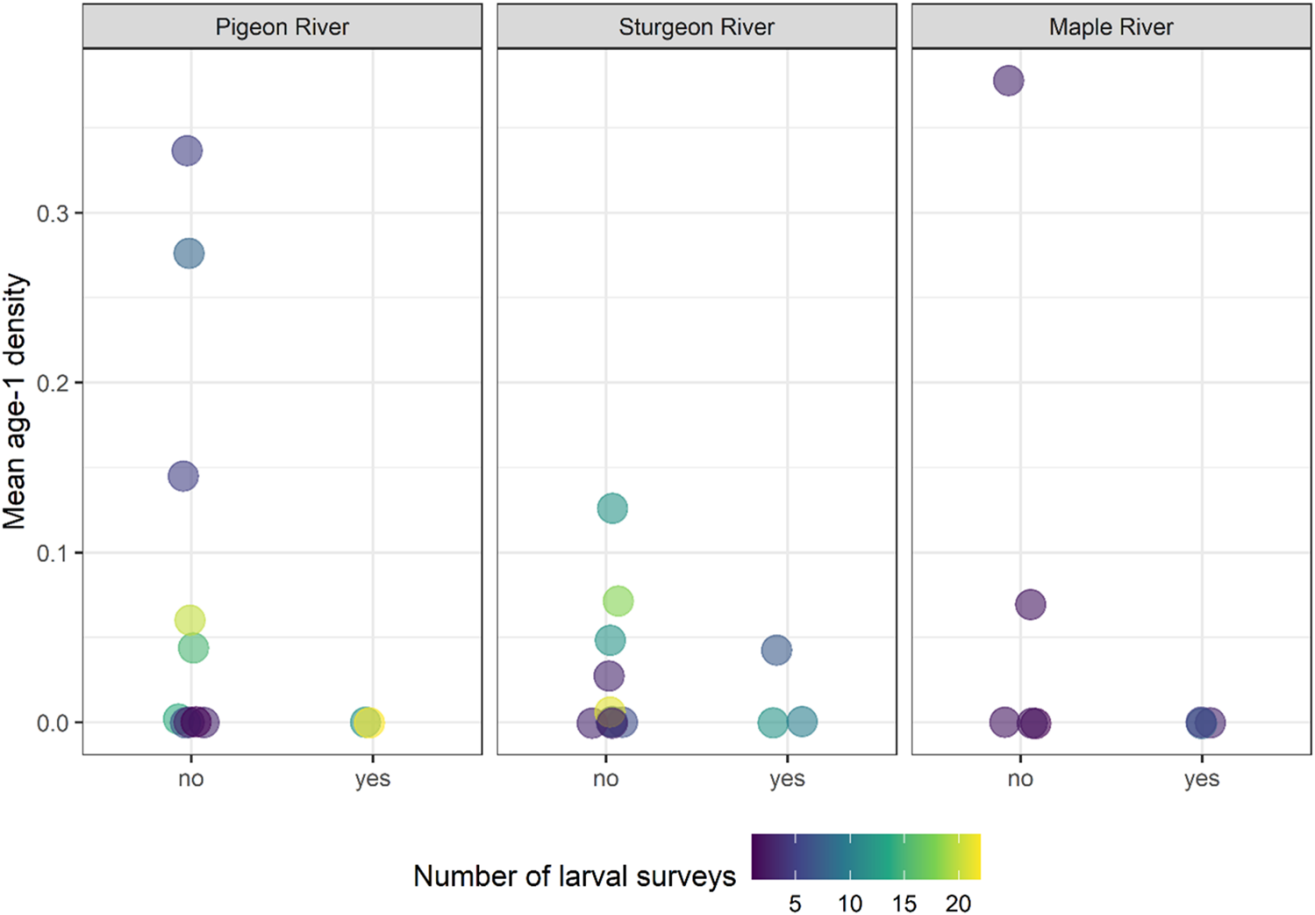
The mean number of age-1 larval sea lamprey per m^2^ of habitat surveyed (Mean age-1 density) during years with (yes) and without (no) the release of sterile male adult sea lamprey. Point color illustrates the number of surveys completed each year and is relative indicator of the confidence in the density estimate.

**Figure 4:**
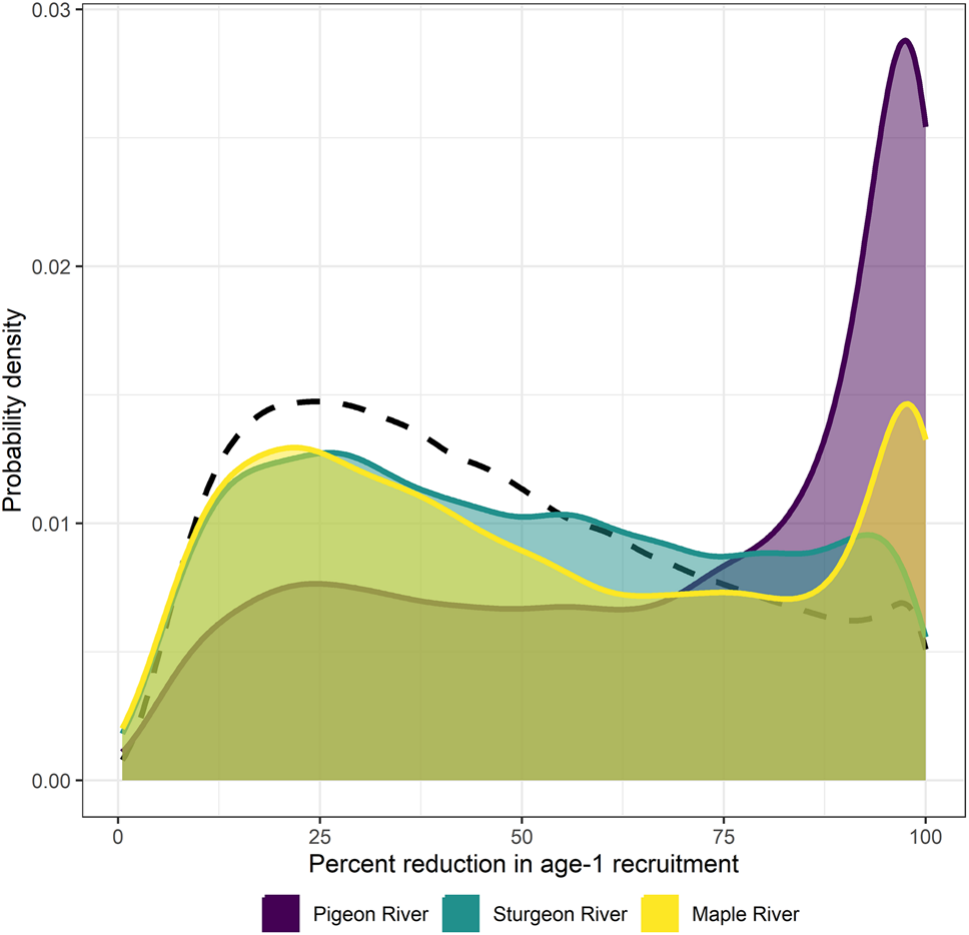
Estimated effect of sterile-male-release technique on age-1 recruitment in the Pigeon, Sturgeon, and Maple Rivers. The prior probability distribution (estimated effect size assigned as input into our Bayesian analysis; broken line with unshaded area-under-curve) and posterior probability distributions (output from our Bayesian analysis; shaded area-under-curve with solid lines) for expected percent reduction in age-1 recruitment associated with releasing 1000 sterile males are depicted. Expected percent reduction is derived from model parameter *q* as 100 * [1 − e^−qE^].

Importantly, the ability to document reductions in recruitment of age-1 larval sea lamprey during years sterile males were released had limited statistical power and estimates of sterile male release effect size had low precision. Recruitment of age-1 larvae varied substantially from year-to-year, independent of whether sterile males were released, and larvae were spatially clustered (non-independent; Figs. 2 and 3, Supplemental Table 3). Releasing 1000 sterile males (roughly what was needed to exceed 35:1 ratio most years) was associated with a 67% reduction in age-1 recruitment in the Pigeon River (95% credible interval: 9–99.9%), a 50% reduction in the Sturgeon River (95% credible interval: 7–98%), and a 52% reduction in the Maple River (95% credible interval: 7–99.9%; Fig. 4).

## Discussion

To our knowledge, this is the first invasive fish population in which the abundance of offspring was documented to decrease over multiple years after the release of sterilized males. In our case, the outcome was delayed lampricide treatment in the Pigeon, Sturgeon, and Maple Rivers because the number of juvenile sea lamprey that would be targeted did not justify the cost of lampricide treatment^27^. The postponement of lampricide treatments in the Pigeon, Sturgeon, and Maple Rivers had the added benefits of allowing resources to be diverted to control sea lamprey in other priority areas in the Great Lakes^12^, thereby collectively benefiting the entire program. The results were not surprising because sterile males were released at ratios of 35:1 or greater on average, immigration rates of new conspecifics were low, and sterile males survived, migrated to spawning habitat, and competed for mates^2,4,23^—a scenario which previous modeling approaches predicted large theoretical reductions in recruitment^25,30^,—and a scenario previous field tests of sterile sea lamprey release were unable to achieve^23^.

Documenting a precise and mechanistic understanding of how sterile male release influenced sea lamprey recruitment was challenging here and could be for other studies. In our case, statistical power was limited (Fig. 4) by the unstructured method by which larval recruitment data were collected in years prior to the release of sterile males, the uncertainty with length-based age assignment in larval sea lamprey^31^, changing stream habitat (removal of two dams in the system)^26^, and the inherent variability in fish recruitment relationships (including sea lamprey)^32^. What is especially unclear is if age-1 recruitment rate is density dependent^33^, a key condition for consistent reductions in recruitment after sterile male release^2^. To address these uncertainties, we initiated an adaptive assessment monitoring plan in 2020^34^ that leverages systematic annual assessments for larval sea lamprey coupled with pedigree analysis to estimate the effective number of spawners each year and accurately age larval sea lamprey^35^. The adaptive assessment plan will mature by 2031 through continued surveys in the Pigeon, Sturgeon, and Maple Rivers as well as ten other streams in the Great Lakes where sterile males may be released. A primary learning objective is to reduce uncertainty around sea lamprey stock-recruitment dynamics^34^.

Integrating pest control tools that are effective on high density populations (pesticides) with those that are effective on low-density populations (sterilization and pheromone-based) may help alleviate limitations in procuring individuals for sterilization. An immediate and obvious limitation of sterile male release for vertebrate pest control is an inability to propagate or capture the large number of sterile individuals needed to achieve overflooding rates. Here, about 1000 sterile males were released per year into each study stream. Over 300 tributaries to the Great Lakes require regular lampricide treatment^29^ and roughly 20,000 adult males are captured in the Great Lakes each year^36^. Therefore, sole reliance on sterile male release as a control tool has exceptionally limited geographic applicability for sea lamprey control.

Sterile male release may find broader application for sea lamprey control and other vertebrate pests if used in combination with tactics that are effective against high density populations (pesticides) and tactics that are effective against low density populations such as removal in traps and pheromone-based approaches^12^. In our study, regular lampricide treatment prior to sterile male release reduced adult sea lamprey density in the Pigeon, Sturgeon, and Maple Rivers^27^ and allowed overwhelming rates of 35:1 to be achieved. Combining sterile male release with additional tactics that remove sea lamprey, would again lower the number of sterile males needed. For example, if 80% of the adult sea lamprey were removed in traps, only 200 sterile males would be needed to reach overflooding rates in the Pigeon River. If 20,000 males can be sterilized per year^23^ and 200 sterile males are needed per stream, integrated approaches to controlling low density populations may be applicable to as many as 30% of sea lamprey producing tributaries.

Therefore, control tools developed for a pest species could be viewed as complementary ‘courses’ in a complete ‘meal’ when deployed sequentially; first with tools that are highly effective at controlling dense populations and then, those that are effective at controlling low density populations^12^. In the case of sea lamprey control, tools effective for high density populations are the only tools currently used (concrete barriers are designed to block 100% of adult populations; lampricides kill 95% of larval populations), leaving opportunities where tools that are effective on low density populations like trapping^37^, chemosensory cues^38^, and seasonal non-physical barriers^39^ could be combined with sterile male release. While these ideas are not new^11^, they have often been overlooked in sea lamprey control during the past 20 years^12^—meanwhile being deployed and shown effective for insect pests such as the Mediterranean fruit fly, screw worm, and tsetse flies^40^.

These findings are timely since the technological and financial hurdles to developing new methods to sterilized vertebrates may diminish in coming years^41–43^. Sea lamprey are currently chemosterilized in a contained laboratory using highly specialized protective equipment^20^, which limits the geographic scope of its deployment, increases labor, facility, and animal transportation costs, and applicability to other pests. RNA interference-based (RNAi) methods for sterilizing are quickly developing for insect pests^44^ and may also be applicable for the relatively small group of fishes that die after spawning like sea lamprey and semelparous salmonids^45^. RNAi-based methods can be species-specific and target specific genes, and therefore, may pose minimal risk to people or the environment^46^. RNAi has already been tested in the laboratory as a next generation sea lamprey pesticide^47^, and similar work could develop the next generation sterilant used to sterilize sea lamprey streamside^14^. The same concept may be applicable to a broad range of vertebrate pests depending on their life history and mating system.

## Methods

### Demographics of adult sea lamprey in the Pigeon, Sturgeon, and Maple Rivers

The abundance, size, fecundity, and migration timing of adult sea lamprey in the Pigeon, Sturgeon, and Maple Rivers 2011–2019 have been previously described^26,27^. Here, using the same methods, adult sea lamprey abundance, size, fecundity, and migration timing in the Pigeon, Sturgeon, and Maple Rivers 2020–2022 was estimated. Notable changes to the study system during the decade-long study include the removal of two dams. The Song of the Morning Dam was removed from the Pigeon River during 2015 and increased habitat available for sea lamprey to occupy by 35 km^27^. The Lake Kathleen Dam was removed from the Maple River during 2018 and increased the habitat sea lamprey could occupy by 34 km^27^.

### Procurement and release of sterile males

Male sea lamprey to be sterilized were procured during their upstream migration from traps located on the lower Cheboygan River, Paradise Lake (Carp Lake) outlet, Ocqueoc River, Manistique River, Peshtigo River, and Thessalon River, which are tributaries to northern Lakes Huron or Michigan. Males were sexually immature (pre-spermiated) when sterilized and were distinguished from females based on their swollen dorsal ridge^48^. Males were sterilized by U.S. Fish and Wildlife Service as described in^49^ according to the animal use and care protocols therein. While other sterilization approaches have been investigated^50^, chemosterilization is the only method available for sterilizing sea lamprey at large scale. Experimental protocols involving the handling of fishes were carried out in accordance with United States federal guidelines for care and use of animals and were in accordance with American Fisheries Society, ARRIVE guidelines, and the U.S. Animal Welfare Act of 1970^51^. Sterile males were fin-clipped prior to release so they could be distinguished from non-sterile males (wild) and released into the Pigeon River at East Mullett Lake Road, the Sturgeon River at Burt Lake State Park, and the Maple River at Brutus Road (within 3 km of their confluence with Burt or Mullett Lakes). Since the sterilization program was decommissioned between 2012 and 2016, we used laboratory assays to verify the sterilization method was still effective when this project started in 2017. Sterility was found to be 100% in 2017 and 2018 and 94% in 2021 using procedures outlined in^49,52^ (Supplemental Table 4; staffing limitations precluded sterility checks in 2019, 2022), where a single sterile male and single wild male were used to fertilize 100–500 eggs of a single female and the percent of sea lamprey embryos fertilized by each male surviving to stage-12 was determined^53^.

The observed ratio of sterile males and wild males on sea lamprey nests in the Pigeon, Sturgeon, and Maple Rivers was assessed in 2017 and 2018 according to procedures described in^23^. Specifically in this study, each year search effort was deployed over about 30 days and 6 h per day during the spawning season and in locations throughout the watershed where spawning habitat was present (less than 1 m in depth, rock substrate 2–5 cm in diameter, water velocity around 0.5 m/s) and larval sea lamprey were found historically. Two field staff would walk upstream and when a sea lamprey spawning nest was identified, would visually determine if sea lamprey were present. Regardless of if sea lamprey were present in the nest, the nest was probed and any embryos or recently hatched larvae present were collected using a drift net set downstream of the nest, counted, and then assessed for viability as described in^23^. While we could not be sure we investigated all possible areas of spawning, we were confident that the sampling covered a representative sample of spawning habitat at times of the year sea lamprey were spawning.

### Estimates of sea lamprey recruitment to age-1 and lampricide treatment decision process

Backpack electrofishing deployed by trained U.S. Fish and Wildlife Service staff was used to capture larval sea lamprey following ranking survey methods described in^54^ with the assumed effectiveness described in^55^. Age-1 and older larvae were targeted because they are easier to see and capture than age-0 larvae, therefore, minimizing inter-personnel differences in catch rates. Differences in water conductivity and depth were assumed not to vary enough with each stream to change electrofishing effectiveness across years^55^. Captured sea lamprey were measured in length and larvae were assumed to have been spawned the previous year (age-1) if they were 20–40 mm and captured in May and June, were 40–60 mm and captured in July and August, and were 60–80 mm and captured in September and October^31^. Lampricide treatments were prescribed for the Pigeon, Sturgeon, and Maple Rivers using the methods in^29^ where treatments only occur when the cost to kill a larval sea lamprey over 100 mm exceeds an economic threshold.

The age-1 recruitment process model was a white noise time series (uncorrelated random variables with a mean of zero) on the log scale with instantaneous mortality proportional to the number of sterile males released (Supplemental Table 3).

$$\log \left( {A_{yr,s} } \right) = a_{s} + a^{\prime}_{yr,s} - q_{s} E_{yr,s}$$

$$a^{\prime}_{yr,s} \sim Normal\left( {0,\sigma_{a} } \right)$$

The log-scale expected recruitment index for stream *s* in year *yr* (*A*_*yr,s*_) is the sum of the average log-scale recruitment rate for stream s (*a*_*s*_) and the deviation from the mean recruitment for year *yr* (*a*′_*yr,s*_) less the instantaneous mortality rate *q* multiplied by the sterile male release control effort (*E* = number of sterile males released/1000). Mortality rate was interpreted on the finite scale and converted to annual percent reduction in recruitment (100 * [1 − *e*^−qE^]). Recruitment index observations (measurements of age-1 density * 1000 rounded to the nearest integer) were assumed to follow a negative binomial distribution, with overdispersion parameter θ allowing for variance to be greater than the expected mean.

$$\widehat{{a_{yr,s,l} }}\sim Negative\;Binomial\left( {A_{yr,s} ,\theta } \right)$$

Model parameters were assigned low information priors (Supplemental Table 3).

Posterior distributions of model parameters were generated through Markov Chain Monte Carlo (MCMC) sampling implemented in Stan^56^ using the rstan package^57^ and R version 4.0.2^58^. Four MCMC chains were run for 5000 iterations with a warmup of 2000 iterations. R-hat statistics calculated for each model parameter (min 1.0, max 1.01) and zero flagged divergent transitions after warmup indicated that saved iterations after the burn-in period were sampling from the posterior distribution and had achieved stationarity.

### Supplementary Information

Supplementary Tables.

## Data Availability

All data generated or analyzed during this study are included in this published article and its Supplemental Information files.
