# Supplementary material for: A decade-long study demonstrates that a population of invasive sea lamprey (Petromyzon marinus) can be controlled by introducing sterilized males
Source: Sci Rep. 2024 Jun 3;14:12689. doi: 10.1038/s41598-024-61460-1 (PMC11148153; doi:10.1038/s41598-024-61460-1)
Supplement: Supplementary file 1 — Supplementary Tables. [file 41598_2024_61460_MOESM1_ESM.pdf]

A decade-long study shows that invasive sea lamprey (*Petromyzon marinus)* can be controlled by the release of sterilized males

Nicholas S. Johnson[1*], Sean A. Lewandoski[2], Aaron K. Jubar[3], Matthew J. Symbal[2], Benson M. Solomon[2], Gale A. Bravener[4], Jessica M. Barber[2], Michael J. Siefkes[5]

[1]USGS, Great Lakes Science Center, Hammond Bay Biological Station, 11188 Ray Road, Millersburg, MI, 49759, USA, 989-734-4768, njohnson@usgs.gov

[2]USFWS, Marquette Biological Station, 1095 Cornerstone Drive, Marquette, MI 49855, USA.

[3]USFWS, Ludington Biological Station, 5050 Commerce Drive, Ludington, MI 49660, USA.

[4]DFO, Sea Lamprey Control Centre, 1219 Queen Street East, Sault Ste. Marie ON P6A 2E5, Canada.

[5]Great Lakes Fishery Commission, 2200 Commonwealth Blvd., Suite 100. Ann Arbor, MI 48105, USA.

*****Corresponding author

Supplemental Table 1. The timing and number of sterile males released in the Pigeon, Sturgeon, and Maple Rivers, Michigan, USA, with the yearly total of sterile males released in each stream each year in bold.

Date	Pigeon	Sturgeon	Maple
22-May-17	400	400	200
23-May-17	250	250	175
24-May-17	200	200	125
25-May-17	200	200	125
31-May-17	125	125	75
1-Jun-17	125	125	75
14-Jun-17	125	125	55
**2017**	**1425**	**1425**	**830**
25-May-18	656	680	367
1-Jun-18	550	550	326
6-Jun-18	75	75	75
12-Jun-18	25	25	25
20-Jun-18	32	20	20
**2018**	**1338**	**1350**	**813**
1-Jun-19	75	225	150
3-Jun-19	187	428	293
11-Jun-19	40	95	65
18-Jun-19	30	90	65
25-Jun-19	28	70	55
**2019**	**360**	**908**	**628**
31-May-21	195	0	0
1-Jun-21	0	0	326
2-Jun-21	0	228	0
3-Jun-21	80	280	320
16-Jun-21	0	20	20
**2021**	**275**	**528**	**666**
25-May-22	445	1150	1140
1-Jun-22	55	210	210
8-Jun-22	25	40	50
**2022**	**525**	**1400**	**1400**

Supplemental Table 2. The location, number, and timing of sea lamprey adult trap deployments in the Pigeon, Sturgeon, and Maple Rivers, MI, USA, 2013-2022, and the number of non-sterile (unmarked) sea lampreys captured.

Stream	Year	Location Road Crossing	Location GPS Coordinates	Number of Traps	Trap ID	Deployment Date	Retrieval Date	Unmarked Lamprey Caught
Pigeon	2013	M-68	45.37433, -84.51502	3 Nets	T1,T2,T3	3-May-13	28-Jun-13	2(unknown net)
Pigeon	2014	M-68	45.37433, -84.51502	3 Nets	T1,T2,T3	24-Apr-14	15-Jul-14	0
Pigeon	2014	Sturgeon Valley Road	45.15616, -84.46782	2 Nets	T1,T2	7-May-14	16-Jun-14	0
Pigeon	2014	Song of The morning dam	45.14607, -84.47284	2 PAT	PAT	7-May-14	16-Jun-14	0
Pigeon	2015	M-68	45.37433, -84.51502	2 Nets	T1,T2	16-Apr-15	8-Jul-15	0
Pigeon	2015	Agnes Andrea Nature Preserve	45.39576, -84.53221	2 Nets	T1,T2	29-Apr-15	8-Jul-15	1(T1)
Pigeon	2016	Agnes Andrea Nature Preserve	45.39576, -84.53221	2 Nets	T1,T2	28-Apr-16	28-Jun-16	0
Pigeon	2016	M-68	45.37433, -84.51502	2 Nets	T1,T2,T3	2-May-23	28-Jun-16	0
Pigeon	2016	Afton Rd	45.36406, -84.50735	2 Nets	T1,T2	15-May-16	28-Jun-16	0
Pigeon	2017	Agnes Andrea Nature Preserve	45.39576, -84.53221	1 Net	T1	14-Apr-17	6-Jul-17	0
Pigeon	2017	M-68	45.37433, -84.51502	2 Nets	T1,T3	24-Apr-17	6-Jul-17	2(T3)
Pigeon	2017	Afton Rd	45.36406, -84.50735	1 Net	T1	24-Apr-17	6-Jul-17	0
Pigeon	2017	Skiera Rd	45.40342, -84.53333	2 Nets	T1,T2	27-Apr-17	6-Jul-17	0
Pigeon	2018	Skiera Rd	45.40342, -84.53333	1 Net	T1	9-May-18	3-Jul-18	0
Pigeon	2018	Afton Rd	45.36406, -84.50735	1 Net	T1	10-May-18	5-Jul-18	1
Pigeon	2018	M-68	45.37433, -84.51502	2 Nets	T2,T3	14-May-18	26-Jun-18 (T2), 5-July-18(T3)	2(T3)
Pigeon	2018	Agnes Andrea Nature Preserve	45.39576, -84.53221	2 Nets	T1,T2	16-May-18	3-Jun-18	0
Pigeon	2018	Skiera Rd	45.40342, -84.53333	1 Net	T2	18-May-18	5-Jul-18	1
Pigeon	2019	Skiera Rd	45.40342, -84.53333	1 Net	T2	3-May-19	2-Jul-19	0
Pigeon	2019	M-68	45.37433, -84.51502	1 Net	T2	6-May-19	9-Jul-19	1
Pigeon	2019	Afton Rd	45.36406, -84.50735	1 Net	T1	6-May-19	8-Jul-19	0
Pigeon	2019	Skiera Rd	45.40342, -84.53333	1 Net	T1	6-May-19	2-Jul-19	0
Pigeon	2020	Skiera Rd	45.40342, -84.53333	2 Nets	T1,T2	7-May-20	17-Jun-20	0
Pigeon	2021	Skiera Rd	45.40342, -84.53333	2 Nets	T1,T2	14-Apr-21	21-Jun-21	2(T1)
Pigeon	2021	M-68	45.37433, -84.51502	2 Nets	T1,T2	14-Apr-21	21-Jun-21	0
Pigeon	2021	Webb Rd	45.27196, -84.45990	1 Net	T1	22-Apr-21	23-Jun-21	7(T1)
Pigeon	2022	Webb Rd	45.27196, -84.45990	1 Net	T1	5-May-22	30-Jun-22	4
Pigeon	2022	Afton Rd	45.36406, -84.50735	1 Net	T1	6-May-22	24-Jun-22	0
Pigeon	2022	M-68	45.37433, -84.51502	2 Nets	T1,T2	5-May-22	30-Jun-22	10(T1)
Pigeon	2022	Skiera Rd	45.40342, -84.53333	2 Nets	T1,T2	5-May-22	24-June-22(T1) 30-June-22(T2)	1(T2)
Sturgeon	2013	Haakwood State Park/ Scott Road	(45.30298, -84.6143), (45.29133, -84.61439)	4 Nets	T1,T2,T3,T4	3-May-13	28-Jun-13	1(unknown net)
Sturgeon	2014	Haakwood State Park/ Scott Road	(45.30298, -84.6143), (45.29133, -84.61439)	4 Nets	T1,T2,T3,T4	25-Apr-14	15-Jul-14	0
Sturgeon	2015	Haakwood State Park	45.30298, -84.61439	2 Nets	T1,T2	16-Apr-15	28-Jun-15	0
Sturgeon	2015	White Rd	45.33850, -84.62753	1 Net	T1	6-May-15	8-Jul-15	0
Sturgeon	2016	South White Rd	45.37196, -84.62429	2 Nets	T1,T2	9-May-16	27-Jun-16	0
Sturgeon	2016	Haakwood State Park	45.30298, -84.61439	1 Net	T2	28-Apr-16	27-Jun-16	0
Sturgeon	2016	Haakwood State Park	45.30298, -84.61439	1 Net	T1	29-Apr-16	27-Jun-16	0
Sturgeon	2016	Fisher woods	45.38953, -84.61693	3 Nets	T1,T2(double)	10-May-16	27-Jun-16	0
Sturgeon	2017	White Rd	(45.33850, -84.62753), (45.37196, -84.62429)	2 Nets	T1,T2	26-Apr-17	29-Jun-17	0
Sturgeon	2017	Haakwood State Park	45.30298, -84.61439	2 Nets	T1,T2	4-May-17	26-June-17(T2), 5-July-17(T1)	0
Sturgeon	2017	Fisher woods	45.38953, -84.61693	2 Nets	T1,T2	8-May-17	5-Jul-17	1(T1)
Sturgeon	2018	Haakwood State Park	45.30298, -84.61439	2 Nets	T1,T2	14-May-18	5-Jul-18	1(T1)
Sturgeon	2018	White Rd	(45.33850, -84.62753), (45.37196, -84.62429)	2 Nets	T1,T2	17-May-18	25-Jun-18	0
Sturgeon	2018	Fisher woods	45.38953, -84.61693	2 Nets	T1,T2	17-May-18	3-Jul-18	0
Sturgeon	2019	Fisher woods	45.38953, -84.61693	2 Nets	T1,T2	9-May-19	5-Jul-19	0
Sturgeon	2019	Haakwood State Park	45.30298, -84.61439	2 Nets	T1,T2	14-May-19	8-Jul-19	2(T1), 2(unknown trap)
Sturgeon	2019	White Rd	(45.33850, -84.62753), (45.37196, -84.62429)	2 Nets	T1,T2	16-May-19	10-Jul-19	1(T1), 2(unknown trap)
Sturgeon	2020	White Rd	45.37196, -84.62429	2 Nets	T1,T2	5-May-20	17-Jun-20	0
Sturgeon	2021	White Rd	45.37196, -84.62429	1 Net	T1	22-Apr-21	22-Jun-21	0
Sturgeon	2021	Haakwood State Park	45.30298, -84.61439	1 Net	T1	22-Apr-21	22-Jun-21	0
Sturgeon	2022	White Rd	45.37196, -84.62429	1 Net	T1	4-May-22	27-Jun-22	3(T1)
Sturgeon	2022	Haakwood State Park	45.30298, -84.61439	2 Nets	T1	19-May-22	24-Jun-22	0
Maple	2013	Maple River Rd	45.50777, -84.76235	2 Nets	T1,T2	12-Apr-13	28-Jun-13	1(unknown net/location)
Maple	2013	Lake Kathleen Dam	45.52899, -84.77536	2 Nets/2 PAT traps	T1(2 PAT's at dam) T2,T3	12-Apr-13	28-Jun-13	0
Maple	2014	Maple River Rd	45.50777, -84.76235	2Nets	T1,T2	1-Apr-14	16-Jul-14	0
Maple	2014	Lake Kathleen Dam	45.52899, -84.77536	2 PAT traps	T1(2 PAT's)	20-May-14	15-Jul-14	2(caught off nests)
Maple	2014	Lake Kathleen Dam	45.52899, -84.77536	2 Nets	T2,T3	22-May-14	15-Jul-14	0
Maple	2015	Maple River Rd	45.50777, -84.76235	1 Net	T1	21-Apr-15	29-Jun-15	0
Maple	2015	Maple River Rd	45.50777, -84.76235	1 Net	T2	22-Apr-15	22-Jun-15	0
Maple	2015	Lake Kathleen Dam	45.52899, -84.77536	2 Nets/2 PAT traps	T1,T2,T3(PAT),T4(PAT)	28-Apr-15	22-June-15(T1)8-July-15(rest of traps)	2(caught off nests)
Maple	2016	Lake Kathleen Dam	45.52899, -84.77536	2 Nets	T1,T2	22-Apr-16	29-Jun-16	0
Maple	2016	Maple River Rd	45.50777, -84.76235	2 Nets	T1,T2	22-Apr-16	29-Jun-16	0
Maple	2017	Maple River Rd	45.50777, -84.76235	2 Nets/2 PAT traps	T1,T2,T3(PAT),T4(PAT)	10-Apr-17	6-July-16(2PAT's) 7-July-17(Nets)	0
Maple	2017	Lake Kathleen Dam	45.52899, -84.77536	1 Net	T1	10-Apr-17	7-Jul-17	0
Maple	2017	Lake Kathleen Dam	45.52899, -84.77536	1 Net	T2	11-Apr-17	7-Jul-17	0
Maple	2018	Maple River Rd	45.50777, -84.76235	1 Net	T2	30-Apr-18	26-Jun-18	0
Maple	2018	Lake Kathleen Dam	45.52899, -84.77536	1 Net	T1	15-May-18	5-Jul-18	0
Maple	2018	Maple River Rd	45.50777, -84.76235	1 Net	T1	16-May-18	2-Jul-18	0
Maple	2018	Lake Kathleen Dam	45.52899, -84.77536	1 Net	T2	16-May-18	26-Jun-18	0
Maple	2018	Lake Kathleen Dam	45.52899, -84.77536	2 PAT traps	T3/T4	21-May-18	2-Jul-18	0
Maple	2019	US31 Bridge	45.54006, -84.78328	1 Net	T1	26-Apr-19	10-Jul-19	0
Maple	2019	Douglass Lake	45.57266, -84.74541	1 Net	T1	3-May-19	5-Jul-19	0
Maple	2019	Mills Rd	45.55096, -84.79652	1 Net	T1	8-May-19	10-Jul-19	0
Maple	2019	Wooldland Rd	45.52899, -84.77536	2 Nets	T1,T2	16-May-19	10-Jul-19	0
Maple	2020	Maple River Rd	45.50777, -84.76235	2 Nets	T1,T2	7-May-20	14-May-20	1(T1)
Maple	2020	Woodland Rd	45.52899, -84.77536	2 Nets	T1,T2	14-May-20	17-Jun-20	0
Maple	2021	Woodland Rd	45.52899, -84.77536	2 Nets	T1,T2	15-Apr-21	23-Jun-21	1(T2)
Maple	2021	US31 Bridge	45.54006, -84.78328	1 Net	T1	15-Apr-21	22-Jun-21	0
Maple	2022	Woodland Rd	45.52899, -84.77536	2 Nets	T1,T2	4-May-22	27-Jun-22	0
Maple	2022	US31 Bridge	45.54006, -84.78328	1 Net	T1	4-May-22	27-Jun-22	0

ile Release Locaitons

Supplemental Table 3. Parameter descriptions, symbols, and prior distributions for the age-1 recruitment and sterile male release effect estimation model.

Parameter Description	Parameter Symbol	Prior Distribution	Posterior Distribution (mean and 95% credible interval)
mean log scale age-1 recruitment index (Pigeon River)	a_{Pigeon}	~Normal (4,5)	-3.38 (-5.78,-1.42)
mean log scale age-1 recruitment index (Sturgeon River)	$a_{Sturgeon}$	~Normal (4,5)	-4.26 (-6.57, -2.43)
mean log scale age-1 recruitment index (Maple River)	a_{Maple}	~Normal (4,5)	-3.36 (-6.68, 0.15)
standard deviation of annual recruitment deviations	σ_a	~Half-Normal (0,5)	2.20 (0.27, 4.48)
mortality rate from SMRT application (Pigeon River)	q_{Pigeon}	log(q) ~ Normal(-0.6,1)	0.65 (-1.84, 2.34)
mortality rate from SMRT application (Sturgeon River)	$q_{Sturgeon}$	log(q) ~ Normal(-0.6,1)	-0.15 (-2.25, 1.52)
mortality rate from SMRT application (Maple River)	q_{Maple}	log(q) ~ Normal(-0.6,1)	0.13 (-2.27, 2.44)
overdispersion parameter of the negative binomial distribution	Θ	~Exponential (1)	0.0026 (0.00147, 0.0041)

Supplemental Table 4. Percent of eggs spawned from females (Females Spawned) that were fertilized with milt from non-sterilized males and sterilized males and survived to developmental stage 12 each year.

Year	Females Spawned	Non-sterile Male Embryo Survival	Sterile Male Embryo Survival
2017	16	65.1%	0.0%
2018	11	38.7%	0.0%
2021	21	34.1%	6.0%
